# The Impact of COVID-19 on the Health-Care Workforce: from Heroes to Zeroes?

**DOI:** 10.2967/jnumed.120.251785

**Published:** 2020-08

**Authors:** Johannes Czernin

**Affiliations:** David Geffen School of Medicine at UCLA, Los Angeles, California

The economic impact of coronavirus disease 2019 (COVID-19) reaches far beyond the business of nuclear medicine and radiology (1–3). The U.S. health-care economy was projected to suffer dramatic financial losses totaling more than $200 billion just for the 4 mo from March through June 2020 (4).

Mass layoffs frequently mitigate the financial stresses of industries. The health-care industry is no exception. Job losses will be most extensive and painful among the low-income and minority populations that also have the highest COVID-19–associated infection and mortality rates. These demographic and economic consequences have not changed much since the 1918 Spanish flu, which also affected low-income populations most gravely.

Hospitals that were already financially stressed before the pandemic began are at highest risk of financial collapse now. These smaller, rural centers provide underserved populations with critical access to essential medical care. Many of these facilities will not survive COVID-19, thus further limiting medical care to already underserved populations. Larger systems will merge with smaller hospitals, and some small clinics will simply disappear.

By the end of April 2020, the ambulatory health-care workforce had dropped by 1.2 million (or >15%), as reported by the Bureau of Labor Statistics. A considerable number of jobs will be regained after an unknown recovery period. However, hospitals cannot spend money they do not have. The government initiated the Coronavirus Aid, Relief, and Economic Security Act, which delivered nearly instantaneous but insufficient financial relief. Much more needs to be done.

The health-care workforce much appreciates flyovers, ticker-tape parades, shout-outs of thank-you, symphonic fanfare, clapping, banging of pots and pans, and many other acts of love that provide a morale boost. But what comes next? Who will help the heroes when their finances drop to zero? Who will help them when they lose not only jobs but also health insurance?

In their current analysis, LoGiudice et al. (1) from Memorial Sloan Kettering state that over the long term, “once fear has subsided, unemployment and insurance coverage will play an important and longer-lasting role.” They quote predictions that “a 15% unemployment rate will translate to an 11% decrease in the number of people covered by employer-sponsored insurance plans, or 17.1 million people.” In a less optimistic model, the outcomes are even more sobering. The changes in insurance coverage, or in other words, in payer mix, will have a detrimental impact on hospital-system balance sheets, which in turn may lead to further furloughs, salary freezes, and layoffs.

LoGiudice et al. point out that the main source of hospital expenses is staff compensation. Obviously, hospitals have little choice but to cut major expenses when the funds supporting salaries are no longer available. The Memorial Sloan Kettering group concludes correctly that in the long run, “companies cannot save their way out of a crisis.” To succeed, health-care systems need to continue to grow and innovate to support job growth rather than contract.

In the interim, because hospitals will be unable to maintain their workforce, governments need to step in to mitigate the devastating job losses in health care. Bailouts have saved the financial sector, the car industry, mortgage lenders, and many more throughout history. The health-care economy needs a major bailout to ensure that hospitals remain solvent, that low- and medium-income workers are not losing their jobs and insurance, and that high-quality health care can be guaranteed.
